# Supplementary material for: Specific polar subpopulations of astral microtubules control spindle orientation and symmetric neural stem cell division
Source: eLife. 2014 Jul 4;3:e02875. doi: 10.7554/eLife.02875 (PMC4112548; doi:10.7554/eLife.02875)
Supplement: Figure 7—source data 1. — DOI: http://dx.doi.org/10.7554/eLife.02875.014 [file elife02875s001.pdf]

Supplementary File 1

Table with the values for the graphs in Figure 7: Key spindle and cell features in neural progenitors are not altered by 30 pM nocodazole.

	**Spindle area (μm^2)**		**Normalized fluorescence intensity**	
	Tis21::GFP–	Tis21::GFP+	Tis21::GFP–	Tis21::GFP+
**no treatment**	38.44±2.68	35.68±1.40	0.72±0.062	0.66±0.046
**Control**	36.87±1.69	36.56±2.34	0.69±0.064	0.67±0.057
**Noc 30 pM**	35.17±2.40	35.68±2.53	0.70±0.058	0.68±0.050
	**Normalized standard deviation of fluorescence intensity**		**Cell diameter (μm)**	
	Tis21::GFP–	Tis21::GFP+	Tis21::GFP–	Tis21::GFP+
**no treatment**	0.62±0.025	0.61±0.029	6.69±0.37	7.10±0.29
**Control**	0.61±0.043	0.63±0.030	8.12±0.35	8.01±0.62
**Noc 30 pM**	0.60±0.037	0.58±0.014	6.86±0.48	7.79±0.69

Values are given as mean±SEM.
